# Chronic Hepatitis C: Acute Exacerbation and Alanine Aminotransferase Flare

**DOI:** 10.3390/v15010183

**Published:** 2023-01-08

**Authors:** Tatsuo Kanda, Naoki Matsumoto, Tomotaka Ishii, Shuhei Arima, Shinji Shibuya, Masayuki Honda, Reina Sasaki-Tanaka, Ryota Masuzaki, Shini Kanezawa, Tsukasa Nishizawa, Yasuhiro Gon, Masahiro Ogawa, Hirofumi Kogure

**Affiliations:** 1 Division of Gastroenterology and Hepatology, Department of Medicine, Nihon University School of Medicine, Itabashi-ku, Tokyo 173-8610, Japan; 2 Division of Respiratory Medicine, Department of Medicine, Nihon University School of Medicine, Itabashi-ku, Tokyo 173-8610, Japan

**Keywords:** acute-on-chronic liver failure, acute liver failure, DAA, flare up, HCV, acute exacerbation

## Abstract

The hepatitis C virus (HCV) causes acute and chronic hepatitis, cirrhosis, and hepatocellular carcinoma, as well as extrahepatic manifestations such as malignant lymphoma. Currently, direct-acting antiviral agents (DAAs) against HCV infection can lead to a sustained virological response (SVR) in almost all HCV-infected patients. In this review article, we discuss acute exacerbation and alanine aminotransferase (ALT) flare in patients with chronic HCV infection. Although acute liver failure caused by HCV infection is rare, careful attention should be paid to the cases with ALT elevation during the natural course of chronic HCV infection. HCV genotype 2 infection, the use of rituximab, and a higher dose of corticosteroid are factors associated with HCV acute exacerbation and ALT flare. Treatment regimens for cancer have been interrupted or changed due to ALT flare due to HCV infection in some patients undergoing chemotherapy for cancer. The pathogenesis of HCV acute exacerbation and ALT flare could involve cellular as well as humoral immune responses. In the DAA era, the earlier introduction of DAAs may prevent chronic HCV-infected patients with acute exacerbation and ALT flare from developing into a more severe form, although DAAs may not be effective for all of them.

## 1. Introduction

The Hepatitis C virus (HCV) was first reported in 1989 [1,2]. Chronic HCV infection causes acute and chronic hepatitis, cirrhosis, and hepatocellular carcinoma (HCC) and is associated with malignant lymphoma and other diseases [3,4]. Approximately 20–25% of patients with chronic HCV infection will progress to cirrhosis over 25–30 years [5]. At 3.8 years after the observation of patients with advanced fibrosis and cirrhosis, the following were observed: an increase of 2 or more points in the Child–Turcotte–Pugh score in 10.9%, the occurrence of HCC in 2.8%, and overall death in 4.6–6.6% [6]. Yokosuka et al. [7] reported that the spontaneous negativation of HCV RNA was 0.3% per year in 320 patients with chronic HCV liver diseases, and all six patients with negativation of HCV RNA had end-stage liver diseases (five with HCC and one with metastatic liver cancer from the colon) and died. Therefore, patients with chronic HCV infection should be treated to eradicate HCV.

Currently, direct-acting antivirals (DAAs) against HCV can create sustained virologic response (SVR) rates with fewer adverse events than interferon-including regimens, which were the previous standard of care [8,9,10,11,12]. Three researchers were the 2020 Nobel Prize winners in physiology or medicine for “the discovery of HCV” [1,2,13]. The recent SVR rates in patients with chronic HCV infection and treatment with DAAs are much higher [10,11,12].

In the DAA era, a very small number of patients fail to achieve an SVR even after DAA treatment [14]. The World Health Organization (WHO) estimated that, globally, approximately 58 million people have chronic HCV infection, with ~1.5 million new infections occurring per year; in 2019, ~290,000 people died from cirrhosis and HCC caused by chronic HCV infection [15]. As ~80% of people do not show any symptoms following the initial infection of HCV, an important problem is that most HCV carriers are unaware of their HCV infection. Currently, no effective vaccine against HCV is available [16,17].

In this review article, acute exacerbation and/or alanine aminotransferase (ALT) flare associated with HCV infection and their treatments are described. Data from published articles regarding “HCV and fulminant hepatitis, acute liver failure (ALF), acute-on-chronic liver failure (ACLF), acute exacerbation, or alanine aminotransferase (ALT) flare” were identified and selected from PubMed and reviewed. We demonstrate the previous studies of HCV-associated ALF, acute exacerbation, and ALT flare and discuss their current problems in HCV-infected patients during cancer chemotherapy and their mechanism and treatment. We also discuss hepatitis B virus (HBV) reactivation in patients treated with DAAs for HCV infection. We expect that this review will help clinicians see HCV-infected patients with acute exacerbation and ALT flare in the DAA era.

## 2. Acute Liver Failure (ALF) and Infection with Hepatitis C Virus (HCV)

Over 16 years (1986 to 2001), a total of 263 patients with acute hepatitis, including ALF, who were admitted to Chiba University Hospital, Chiba, Japan, were investigated. HCV caused acute hepatitis, non-severe or severe types, and ALF with hepatic coma in 39 (30%) of 129 or 1 (2%) of 52, and none (0%) of 82 patients, respectively [18]. HCV infection was a rare cause of ALF. These facts are notable because HCV is a major cause of cirrhosis and HCC in Japan compared to other Asian countries [19].

Definition, risk factors, natural history, and treatment of acute hepatitis C were well documented in the previous reports [20]. HCV RNA was detected in the earlier serum samples, 9–11 weeks before anti-HCV C100-3 antibodies, and this may be valuable as a diagnostic marker for acute hepatitis C [21]. In patients with acute post-transfusion hepatitis C, overt icteric or higher ALT levels of more than 1000 IU/L are less observed [22,23], although symptoms may include malaise, weakness, anorexia, and jaundice [20]. However, HCV can lead to persistent infection in a high proportion of infected individuals [20].

In general, HCV-related ALF rarely occurs worldwide [18,23,24,25,26,27,28,29,30,31,32,33,34,35,36,37,38,39,40,41,42,43,44,45]. However, the HCV cell-culture-grown virus JFH1 is derived from a Japanese fulminant hepatitis patient [46,47]. Acetaminophen overdose is the leading cause of ALF in the United States [48], and HCV infection may exacerbate acetaminophen-induced liver failure [49]. Uehara et al. observed that HCV transgenic mice expressing the HCV core, E1 and E2, exhibited signs of liver mitochondria dysfunction, showing a potential mechanism for increased susceptibility to acetaminophen [50]. Ramachandran et al. reported that in transgenic mice expressing the HCV core, E1 and E2 proteins amplified mitochondrial oxidant stress in acetaminophen-induced liver injury [51].

In Taiwan, 288 patients who underwent cardiovascular surgery and received blood transfusion were followed prospectively with serum liver aminotransferase levels and viral hepatitis markers for at least six months [24]. Of the 34 non-A non-B post-transfusion hepatitis patients, 15 (44.1%) were asymptomatic, 16 (47.1%) showed clinical symptoms, and 9 (26.5%) showed serum total bilirubin levels higher than 2 mg/dL. There was no case of fulminant hepatic failure [24]. Wright et al. have demonstrated that HCV does not cause fulminant non-A, non-B hepatitis in the United States [25]. Theilmann et al. also concluded that fulminant and subacute hepatic failure is induced by HCV only in few German patients with non-A, non-B hepatitis [27]. Thus, HCV-related ALF rarely occurs [18,23,24,25,26,27,28,29,30,31,32,33,34,35,36,37,38,39,40,41,42,43,44,45]; however, careful attention should be paid to some patients with severe presentation [52,53,54,55,56,57,58,59,60,61,62,63,64].

## 3. Acute Exacerbation and Alanine Aminotransferase (ALT) Flare of Chronic Hepatitis C Virus (HCV) Infection

In the first few weeks after acute initial HCV infection, anti-HCV antibodies might not be detected in patient’s sera, although HCV RNA can be detected. During acute initial HCV infection, seroconversion from negative to positive of the anti-HCV antibodies is also observed. The presence of anti-HCV antibodies indicates a current or previous HCV infection [20]. In general, positivity for both anti-HCV antibodies and HCV RNA indicates acute exacerbation in patients with ALT flare and chronic HCV infection. The definition of acute exacerbation and ALT flare of chronic HCV infection was wide variety [65,66,67,68,69].

Hiraga et al. observed 22 patients (1.3%) with serum ALT flare > 500 IU/L among 1760 patients with chronic hepatitis C between August 1969 and August 2002 [65]. They reported that the HCV genotype 2 infection was the only significant determinant of ALT flare (*p* = 0.0033) using multivariate analysis [65]. Rumi et al. also investigated 100 patients with the HCV genotype 2c and 106 with the HCV genotype 1b chronic hepatitis during a period of 71 (24–144) months [66]. They found 39 (18.9%) with chronic hepatitis reactivation, which they defined as ALT > 400 IU/L or ALT > 8-fold upper limit of normal (ULN), among 206 patients: 7.5% (8/106) and 31% (31/100) belong to HCV genotypes 1b and 2c, respectively [29]. The HCV genotype 2c infection was the significant determinant of chronic hepatitis reactivation (odds ratio (OR) 6.48 (95% confidence interval (CI) 2.57–16.35)) [66]. They also observed that patients with ALT flare and no treatment showed progression in liver fibrosis in repeated liver biopsies after 3–10 years. As such, it is important to eradicate HCV from chronic hepatitis C patients with ALT flare. The various etiologies of ALT flares in patients with chronic hepatitis C are as follows: spontaneous, molecular-targeting therapies (such as antitumor necrotic factor alfa and anti-CD20), the superinfection of HCV, hepatitis A virus (HAV) or HBV, and chemotherapies [67].

Attention should be paid to cases with ALT elevation during the natural course of chronic HCV infection. It has been reported that HCV genotype 2 infection is associated with acute exacerbation [65,66]. It may be difficult to distinguish between acute HCV infection or acute exacerbation and acute hepatitis due to other causes.

## 4. Acute Exacerbation and Alanine Aminotransferase (ALT) Flare among Chronic Hepatitis C Virus (HCV)-Infected Patients during Cancer Chemotherapy

It might not be clear at all whether the anticancer therapy-related elevation of liver enzymes is due to HCV reactivation or infection. Li et al. reported that cancer patients with HCV infection (*n* = 306) showed a higher frequency of severe acute liver injury (2.3% vs. 0.7%; *p =* 0.003) than those without (*n* = 4419) [68]. Li et al. also observed 7 patients (2.3%) with serum ALT flare equal to or more than 400 IU/L among 306 patients with chronic hepatitis C, either during cancer chemotherapy or after 6 months of its cessation [68]. HB surface antigen (HBsAg)-negative and anti-HCV-positive cancer patients with hematological malignancy, HCC, or solid tumors other than HCC, respectively, had 9.32% (3/32), 1.90% (2/105), and 1.18% (2/169) of severe liver injury while receiving chemotherapy [68]. On the other hand, HBsAg-negative and anti-HCV-negative cancer patients with hematological malignancy, HCC, or solid tumors other than HCC, respectively, had 4.10% (22/537), 0.66% (1/151), and 0.24% (9/3731) of severe liver injury when receiving chemotherapy [68]. Among all cancer patients or solid tumors other than HCC patients, patients with anti-HCV are at a higher risk of severe liver injury than those without anti-HCV (*p* = 0.003 or *p* = 0.02, respectively) [68]. Importantly, chemotherapy for cancer was interrupted among 57.1% (4/7) of patients with serum ALT flare. Hematological malignancy and the use of rituximab were identified as risk factors for severe liver injury during chemotherapy. Of interest, none of these patients had liver failure [68].

Torres et al. conducted a prospective observational study of HCV-infected patients receiving cancer treatment between November 2012 and July 2016 [69]. They defined HCV RNA levels > 1 LIU/mL (over baseline) and ALT levels equal to or more than 3-fold ULN as reactivation and hepatic flare, respectively. They observed 23% (23/100) of patients with HCV reactivation: 18 and 5 patients had hematological malignancy and solid tumors, respectively. Of these 23 patients, 10 patients (43%) also had ALT flare. The multivariate analysis demonstrated that the associated factors with HCV reactivation were the use of rituximab (OR, 9.52 (95%CI: 2.19–49.2); *p* = 0.001) or higher-dose corticosteroid (>600 mg equivalent prednisone) (OR, 5.05 (95%CI: 1.40–20.23); *p* = 0.01), and baseline HCV RNA levels of more than 6 LIU/mL (OR, 0.12 (95%CI: 0.03–0.46); *p* < 0.001). After 36 weeks of the commencement of cancer chemotherapy, no liver failure was observed, and no patients died from liver failure [69]. Treatment regimens for cancer were interrupted or changed due to ALT flare as a result of HCV infection in 6 (26%) of 23 patients.

Hematological malignancy, the use of rituximab, and higher doses of corticosteroid were identified as risk factors for severe liver injury when receiving chemotherapy. HCV infection did not prevent the commencement of cancer chemotherapy. Anti-HCV treatment before or during cancer chemotherapy could prevent the interruption of or change in the protocol regimens of cancer chemotherapy [69].

## 5. Novel Anticancer Therapies and Acute Exacerbation and Alanine Aminotransferase (ALT) Flare of Chronic Hepatitis C Virus (HCV) Infection

The risk of HBV or HCV acute exacerbation is not well-defined in cancer patients receiving novel anticancer therapies, such as immune checkpoint inhibitors (durvalumab, atezolizumab, nivolumab, pembrolizumab, ipilimumab, and tremelimumab); BTK inhibitors (ibrutinib and acalabrutinib); agents targeting CD22 (inotuzumab ozogamicin), CD38 (daratumumab, isatuximab), and CC chemokine receptor 4 (CCR4) (mogamulizumab); and chimeric antigen receptor (CAR) T-cell therapy (axicabtagene ciloleucel) [70]. Although HCV acute exacerbation is rare, as only a few cases of HCV acute exacerbation induced by immune checkpoint inhibitors have been reported [71,72,73,74], careful attention should be paid to these HCV-infected patients with cancer (Table 1).

## 6. Mechanism of Acute Exacerbation and Alanine Aminotransferase (ALT) Flare of Chronic Hepatitis C Virus (HCV) Infection

Cellular immune responses play an important role in the pathogenesis of chronic hepatitis C [75,76,77,78,79,80,81,82,83,84,85,86,87,88,89,90,91,92,93]. The secretion of cytokines such as interleukin (IL)-2, interferon (IFN)-γ, and tumor necrosis factor (TNF)-α by CD4^+^ and CD8^+^ T lymphocytes activates antiviral mechanisms [76]. As there have been several reports about the effects of rituximab, a chimeric CD20 monoclonal antibody, on the course of chronic HCV infection, humoral immune responses also play roles in the acute exacerbation and hepatic flare of chronic HCV infection [77,78,79].

Rituximab accelerated HCV replication in patients with non-Hodgkin’s lymphoma (NHL) [80,81,82,83,84,85,86,87]. Lake-Bakarr et al. demonstrated a reduction in HCV hypervariable region 1 (HVR1) sequence diversity with the depletion of B cells in patients with acute exacerbation and ALT flare following rituximab therapies for HCV-related mixed cryoglobulinemia [87]. Thus, humoral immunity also plays an important role in controlling serum HCV RNA levels [87,88].

**Figure 1:**
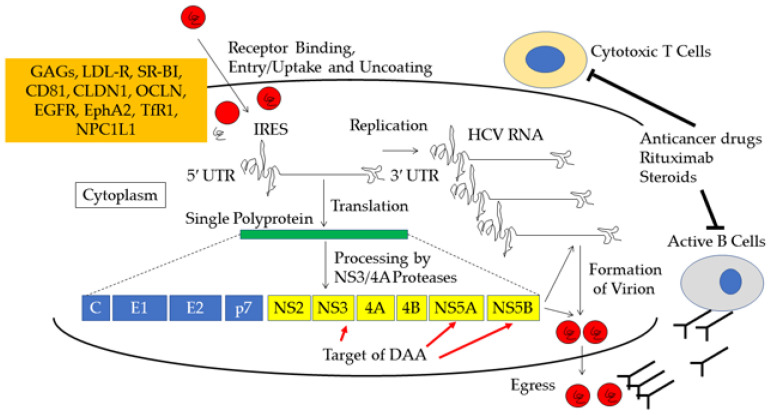
Life cycle of hepatitis C virus (HCV) and mechanism of HCV acute exacerbation and direct-acting antivirals (DAA). Candidates of HCV receptors: glycosaminoglycans, GAGs; low-density lipoprotein receptor, LDL-R; scavenger receptor class B type I, SR-BI; claudin-1, CLDN1; occludin, OCLN; epidermal growth factor receptor, EGFR; ephrin receptor A2, EphA2; transferrin receptor 1, TfR1; Niemann–Pick C1-like 1, NPC1L1. IRES, internal ribosomal entry site; UTR, untranslated region; C, core; E1 and E2, envelope glycoproteins 1 and 2; p7, transmembrane protein 7; NS, nonstructural protein. See the references [75,89].

Hiura et al. also reported a case of severe acute hepatitis C and delayed antibody production due to rituximab [75]. These reports suggest the presence of stronger antibody-mediated immune pressure on HCV infection [89,90]. As innate and acquired immune responses play a role in the control of HCV replication [76,91,92,93] in the pathogenesis of the acute exacerbation and hepatic flare of chronic HCV infection, cellular, as well as humoral, immunological responses may be involved (Figure 1).

## 7. Treatment for Acute Exacerbation and Alanine Aminotransferase (ALT) Flare among Chronically Hepatitis C Virus (HCV)-Infected Patients with Cancer

### 7.1. Treatment for Patients with Non-Hodgkin’s Lymphoma (NHL)

Almost all patients infected with HCV are excluded from clinical trials for cancers other than HCC/liver cancer. Specifically, patients with HCV infection, such as those with HCV antibody-positive or viral hepatitis, are excluded [94]. An association between HCV infection and lymphoproliferative disease has been reported [95,96,97]. The prevalence of HCV infection in patients with B-cell non-Hodgkin’s lymphoma was higher than that of patients without it [95]. A meta-analysis of epidemiological studies demonstrated that the OR for NHL was 5.70 (95% CI, 4.09–7.96; *p* < 0.001) [96]. Although a similar trend for B-cell origin (5.04, 95% CI: 3.59–7.06) and T-cell origin (2.51, 95% CI: 1.39–4.56) [96] was also observed, in general, a positive association is apparent between HCV and the risk of NHL, particularly that of a B-cell origin [97]. Thus, a highly positive association between anti-HCV seropositive subjects and the risk of NHL could exist.

Antiviral therapies produce HCV RNA clearance and consequent tumor regression in most patients with HCV-related low-grade NHL. Antiviral therapies used at any time are associated with improved overall survival (OS) [98]. Antiviral therapy with interferon and ribavirin is able to induce a 70–75% response rate in patients with HCV-associated low-grade NHL who do not need immediate conventional treatment [99]. It is possible that a better prognosis could be achieved by performing HCV antiviral therapy after achieving remission in the cases of HCV-RNA-positive diffuse large-cell lymphoma through the use of R-CHOP, which includes rituximab, cyclophosphamide, doxorubicin, vincristine, and prednisone, as well as similar treatments [100]. As such, a SVR after DAA treatment for HCV infection improves the OS and prognosis of NHL patients.

Shiba et al. described a case of ACLF as the presenting manifestation of diffuse large B-cell lymphoma (DLBCL) in an elderly black man with an human immunodeficiency virus (HIV)/HCV co-infection and prior Hodgkin’s disease that had been in remission for three years [101]. Although ACLF, as the presenting feature of DLBCL, is uncommon, it is possible that the HCV infection of B cells might have caused lymphoma and ACLF.

HCV infection among hematopoietic cell transplant donors and recipients has been demonstrated by the American Society for Blood and Marrow Transplantation [102]. HBV as well as HCV infection occasionally causes severe HCV acute exacerbation as an early complication of hematopoietic cell transplantation [103,104]. After a thorough assessment of the potential hematological toxic effects and drug–drug interactions, the choice of regimens with DAAs is individualized, and DAAs for HCV infection are given properly on-demand [105].

### 7.2. Treatment for Patients with Cancer

In general, a SVR achieved by DAA treatment could lead to an improvement in liver function and the prevention of HCV acute exacerbation and ALT flare during and after chemotherapy for cancers in the short term. In the long term, a SVR could lead to the prevention of fibrosing sclerosing hepatitis or cirrhosis, resulting in the prevention of the occurrence of liver failure and HCC and a subsequent improvement in prognoses among HCV-infected patients [76,106]. Higher levels of liver fibrosis in a cohort of veterans with chronic viral hepatitis were associated with extrahepatic cancers [107]. DAA treatment plays an important role in supporting the treatment of patients with extrahepatic cancers. Due to the use of immunosuppressants and/or anticancer agents, liver failure may develop, and a dose reduction of these treatments may be needed in certain cases of cirrhosis and HCV infection. It is possible that sufficient cancer treatment for other organs, as well as the liver, may be performed after the achievement of a SVR.

In cancer patients, liver dysfunction may be caused by something other than HCV infection, such as circulation failure, viral hepatitis, metabolic syndrome, cancer metastasis and direct invasion to the liver, autoimmune liver disease, drug-induced liver injury, or immune checkpoint inhibitor hepatitis [108,109]. In these patients, liver biopsy may not be performed due to hematological disorders. In the interferon era, the SVR rate is higher in patients with an acute exacerbation of HCV infection, and DAA could lead to a SVR in almost all patients [12].

If advanced HCC patients with HCV infection achieve a SVR using interferon and/or DAAs, their liver function could improve, their treatment duration could be longer, their prognosis might be better, and their OS could improve [110]. Any chance to eradicate HCV should not be avoided. HCV screening is recommended among patients undergoing chemotherapy to allow for the close follow-up of ALT flare [111].

### 7.3. Direct-Acting Antivirals (DAAs) Could Support Sufficient Lung Cancer Chemotherapy in a Patient with Hepatitis C Virus (HCV) Infection and Decompensated Cirrhosis

A man in his 70s was diagnosed with lung adenocarcinoma (T1bN2M0IIIB Stage IIIB) with the Eastern Cooperative Oncology Group/World Health Organization Performance Status (ECOG/WHO PS) Grade 1 [112] and HCV-related liver cirrhosis. He received laparoscopic partial gastrectomy for early gastric cancer 10 years ago. He received partial hepatectomy for HCC 6 years ago. He also takes telmisartan daily for his hypertension, and he continues consuming alcohol. Although HCC has not recurred, he has never received DAA treatment for the eradication of HCV.

**Figure 2:**
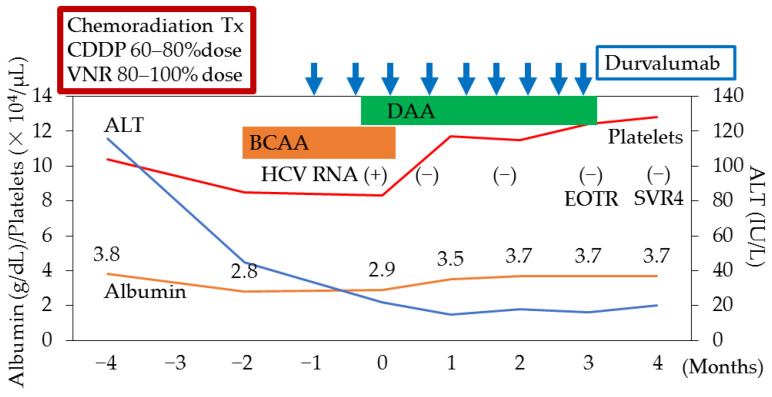
Clinical course of a patient with hepatitis C virus (HCV) infection and decompensated cirrhosis in whom DAA could support sufficient lung cancer chemotherapy. CDDP, cisplatin; VNR, vinorelbine; ALT, alanine aminotransferase; BCAA, branched-chain amino acid; DAA, direct-acting antiviral against HCV; EOTR, end of the treatment response; SVR4, sustained virological response at week 4.

In our outpatient clinic, his HCV RNA levels and HCV genotype were 3.8 LIU/mL and 2a, respectively. His Child–Pugh (CP) grade and score were A and 6, respectively. He began treatment with radiation therapy (60 Gy), combined with chemotherapy (cisplatin (CDDP) (80% dose)/vinorelbine (VNR) (100% dose) and CDDP (60% dose)/VNR (80% dose)) for his lung cancer. At his first visit (−4 months), his aspartate aminotransferase (AST), ALT, albumin, total bilirubin, platelet count, and FIB-4 index [113] were 96 IU/L, 116 IU/L, 3.8 g/dL, 0.83 mg/dL, 10.4 × 10^4^/μL, and 6.51, respectively (Figure 2).

Two and three months later, respectively, he commenced treatment with durvalumab for lung cancer and a combination of sofosbuvir with velpatasvir for his HCV infection and decompensated cirrhosis. One month after the commencement of DAA, the serum HCV RNA was undetectable, and his albumin level changed to 3.5 g/dL. Moreover, a SVR at 24 weeks (SVR24) was achieved and anti-lung-cancer treatment continued (Figure 2). The eradication of HCV could lead to the safe continuation of lung cancer treatment with durvalumab, which is a human immunoglobulin G1 kappa (IgG1κ) monoclonal antibody that blocks the interaction of programmed cell death ligand 1 (PD-L1) with the PD-1 (CD279).

## 8. Hepatitis B Virus (HBV) Reactivation in Patients Treated with Direct-Acting Antivirals (DAAs) for Hepatitis C Virus (HCV) Infection

In general, the development of hepatic failure was less frequent than that of HCC in Japanese patients with chronic HCV infection [114]. It is important for a favorable prognosis among patients with HCV infection to achieve a higher SVR and inhibit the development of HCC. In the DAA era, for HCV infection, SVRs are very high, but close attention must be paid to the possible occurrence of HCC and reactivation of HBV in patients with co-infection who achieve a SVR in the short term. Although HCC occurred in ~30% of patients with a previous HCC history within 15.4 months mean follow-up post-DAA initiation, HCC occurred in only 1.3% of patients without a previous HCC history with 18.2 months mean follow-up [115].

Among HBsAg-positive patients coinfected with HCV, HBV DNA reappearance and reactivation are frequent events until at least 12 weeks after the end of DAA treatment for HCV infection [115,116]. In HBsAg-positive patients, pre-emptive nucleos(t)ide analog treatment should be started to prevent HBV reactivation. A pretreatment HBV DNA level of 300 IU/mL may predict HBV flare and HBsAg seroclearance after anti-HCV therapy [117].

Among HBsAg-negative patients with HCV infection but positive for anti-HBc antibodies and/or anti-HBs antibodies at baseline, HBV reactivation and/or HBV DNA reappearance are rare events until 12 weeks after the end of DAA treatment for HCV infection [115]. When abnormal liver function tests are observed during DAA treatment and after the end of DAA treatment for HCV infection, HCV RNA, HBsAg, and HBV DNA should be examined, and nucleos(t)ide analogs should be used to treat HBV reactivation [115]. The profiles of serum cytokines/chemokines may be useful for the prediction of HBV reactivation [77,118,119]. Among patients coinfected with HBV and HCV, the baseline HBsAg level was also the predictive factor associated with HBV reactivation following DAA treatment [120]. In the DAA treatment for HCV acute exacerbation, attention should be paid to HBV reactivation.

Of interest is the fact that HCV acute exacerbation also occurred during HBV suppression using nucleos(t)ide analogue therapies, and the rates were 4.5% (3/66), 15.4% (2/13), and 1.9% (1/53), for all patients, patients with chronic hepatitis C, and patients with resolved past HCV infection, respectively [121]. An open trial was conducted among four patients who had lost their serum HBsAg following interferon treatment but had continuing HCV viremia and hepatitis [122]. Together, treatments for both HBV and HCV infection, including DAAs, may be considered for patients coinfected with HBV and HCV [115].

Although ALF caused by HCV infection is rare, HCV acute exacerbation occasionally leads to severe hepatitis [123]. One of the limitations is that the number of studies about ALF and acute exacerbation due to HCV is small. Another limitation among these studies is that the definition of HCV acute exacerbation and ALT flare is different between each study. When HCV RNA is positive, DAA treatment should be started. HCV acute exacerbation and ALT flare may not be serious issues in the DAA era, as DAA-based treatment is equally effective in those on immunosuppression such as HIV or other statuses. Most of the currently available DAAs are effective for almost all HCV genotypes [11,12].

DAAs may be effective for the prevention of development into ALF among patients with acute HCV infection [124]. In patients with HCV-related ACLF, compared to previous studies on hepatitis B virus-related ACLF and alcohol-related ACLF, the prevalence of liver failure was very low (17.1%), whereas that of kidney failure was very high (71.4%) [125]. DAAs may not be effective for all patients with HCV infection and ACLF [126]. In the DAA era, however, as HCV infection may occasionally be associated with ACLF, which could lead to poor survival [125,126,127,128,129], we also focused on HCV acute exacerbation and ALT flare in this article. Although there are several excellent reviews about acute exacerbation and alanine aminotransferase flare in HCV carrier patients [77,86], we added the recent literature and reports in this review.

## 9. Conclusions

The previous studies of HCV-associated ALF, acute exacerbation, and ALT flare were demonstrated. We also present a case with HCV infection and decompensated cirrhosis in whom DAAs could support sufficient lung cancer chemotherapy. Careful attention should be paid to cases of ALT elevation during the natural course of chronic HCV infection. The HCV genotype 2 infection, use of rituximab, and higher doses of corticosteroid are factors associated with HCV acute exacerbation. The earlier introduction of DAAs may prevent HCV acute exacerbation from developing into a more severe form, although DAAs may not be effective for all HCV-infected patients with acute exacerbation or ALT flare.

## Figures and Tables

**Table 1:** Hepatitis C virus-infected patients with acute exacerbation and alanine aminotransferase flare during the use of immune checkpoint inhibitors.

Case No.	Age (years) /Sex	HCV RNA (LIU/mL) /HCV Genotypes (GTs)	Max ALT (IU/L)	HIV	Type of Cancer	Drugs /Continued	DAAs for HCV	Refs
1	Unknown	4.55/GT 1a	700–800	Unknown	Unknown	Nivolumab/ Unknown	None	[71]
2	59/Female	6.36/GT 1b	100–120	Unknown	Metastatic melanoma	Pembrolizumab /Yes	LDV/SOF	[72]
3	49/Male	5.94/GT 1c	Elevation (CTCAE grade 1)	Positive	Metastatic melanoma	Pembrolizumab /No due to PD	None	[72]
4	54/Male	Positive/Unknown	Elevation (CTCAE grade 2)	Unknown	Melanoma Stage IV	Pembrolizumab /Yes	LDV/SOF	[73]
5	48/Male	6.3/GT 2a	559	Negative	Squamous cell lung cancer	Nivolumab/No	SOF/RBV	[74]

HCV, hepatitis C virus; ALT, alanine aminotransferase; HIV, human immunodeficiency virus; DAA, direct-acting antivirals; CTCAE, common terminology criteria for adverse events; PD, progressive disease; LDV, ledipasvir; SOF, sofosbuvir; RBV, ribavirin; Refs, references.

## Data Availability

The data underlying this article are available in this article.
